# Lumbar Tractions in Radicular Pain Caused by Herniated Disc: Randomised, Open-Label, Superiority, and Controlled Trial on 424 Participants

**DOI:** 10.3390/jcm14155192

**Published:** 2025-07-22

**Authors:** Elsa Bernhard, Ambre Hittinger-Roux, Helene Delaplace, Loïc Pauvele, Isabelle Charlot, Marion Geoffroy, Lukshe Kanagaratnam, Christophe Eap, Christophe Mensa, Loïs Bolko, Jean-Hugues Salmon

**Affiliations:** 1 Department of Rheumatology, University of Reims Champagne-Ardenne (URCA), Reims University Hospital, 51100 Reims, France; 2 Department of Clinical Research and Public Health, University of Reims Champagne-Ardenne (URCA), University Hospital of Reims, 51100 Reims, France; 3 Department of Neurosurgery, University of Reims Champagne-Ardenne (URCA), University Hospital of Reims, 51100 Reims, France; 4 Department of Orthopedic Surgery, University of Reims Champagne-Ardenne (URCA), University Hospital of Reims, 51100 Reims, France

**Keywords:** radicular pain, lumbar tractions, therapeutics

## Abstract

**Background/Objectives**: Radicular pain is a frequent pathology, and disc herniation is the commonest aetiology. A meta-analysis summarising international guidelines for radicular pain, published in 2021, showed that lumbar traction’s place is still a topic of debate. In this study, our aim was to evaluate the effectiveness of lumbar tractions in treating radicular pain of discal origin in association with medical treatment versus medical treatment alone. We performed a randomised, controlled, interventional, prospective, superiority trial in Reims Hospital Rheumatology Unit. **Methods**: We included participants with radicular pain and concordant disc herniation with ambulatory treatment failure. Participants were randomised into two groups: medical group (analgesics, anti-inflammatories treatments, at least two epidural injections); tractions group with this medical treatment associated with lumbar tractions. The primary outcome was the difference in the proportion of participants experiencing a minimum of 25% improvement in radicular pain at one month follow-up between the two groups. **Results**: We included 424 participants: 211 in the tractions group and 213 in the medical group. We analysed 388 participants (194 in each group). We collected demographic and clinical data, lumbar and radicular Numeric Pain Scale at baseline, one and three months. A statistical difference was found for the primary outcome: 120/194 participants (62%) in tractions group and 98/194 participants (51%) in medical group (*p* = 0.024). **Conclusions**: To our knowledge, this is the first randomised and controlled study on this topic with these results. We can assert the superiority of lumbar tractions in association with medical treatment over medical treatment alone for radicular pain with concordant disc herniation.

## 1. Introduction

Radicular pain is a common condition that originates from the lumbar region and radiates to one or both lower limbs with metameric topography due to nerve damage [1]. Disc herniation represents 85% of their aetiology [2,3]. A herniated disc is an extrusion of the nucleus pulposus resulting in compression of the nerve root [4]. A study has estimated that the lifetime prevalence of radicular pain due to disc herniation is between 13% and 40% [5]. Disc herniation prevalence is higher in the young population, between 25 and 55 years old, 95% of disc herniation is located on the L4/L5 and L5/S1 disc [1,6]. In 2019, French therapeutics guidelines have been published concerning the medical care of acute lumbar pain and acute radicular pain [7]: physical activity and advice to stay active [8], physiotherapy, analgesics treatments, non-steroid anti-inflammatory drugs, and eventually epidural injections. Additionally, a 2021 meta-analysis of international guidelines found inconsistent recommendations regarding lumbar tractions [9]. In France, epidural injections are commonly practiced despite conflicting results in the literature. In 2021, a meta-analysis on 17 studies have shown a trend in clinical improvement compared to placebo [10]. In addition, a 2001 study has shown that epidural injections appear to be effective [11]. Lumbar tractions have been performed empirically for many decades even if the physio-pathological mechanism remains uncertain. However, a few studies have shown a decrease in hernia size on scanner or MRI (Magnetic Resonance Imaging) after lumbar tractions [12,13,14]. In the Rheumatology Unit of Reims Hospital, we use lumbar tractions in association with the usual medical treatment recommended since a few years ago on participants with radicular pain. From our practice, we noticed a trend in improvement in patients’ pain with the combination of the two treatments. In this study, the aim was to evaluate the superiority of lumbar tractions in association with medical treatment compared to medical treatment alone for people with radicular pain.

## 2. Materials and Methods

(a) Design

A prospective, controlled, randomised, interventional, monocentric, and open-label trial was conducted in the Rheumatology Unit at Reims Hospital, France, until 2021. The protocol received approval from the Personal Protection Committee East 1 and all participants provided informed consent. The study was conducted in accordance with the Declaration of Helsinki. Considering our intervention by lumbar tractions, a double-blind study was impossible. We used CONSORT reporting guidelines [15]. This trial has been registered on clinicaltrials.gov with the number NCT02793440 registered in 8 June 2016.

By using a randomisation software, each person was randomly categorised in the medical group (MG) or in the tractions group (TG) with 1:1 ratio per block of 4. Randomisation was performed by the treating physicians responsible for each person, using a software (CleanWeb^®^ Version 178.9.0, Telemedicine Technologies S.A.S., 121 rue d’Aguesseau, 92100, Boulogne-Billancourt, France)

All people received medical treatment (analgesics treatments, anti-inflammatory treatments, physiotherapy, and at least two epidural injections) and tractions group participants benefited with at least three lumbar tractions in addition.

(b) Participants

Patients hospitalised in the Rheumatology Unit in Reims Hospital for an acute, subacute, or chronic radicular pain in failure of ambulatory treatment with a concordant disc herniation (grade II) confirmed on MRI or CT (Computed Tomography) were selected. TheThere was not a defined threshold for pain intensity but in clinical practice the cut-off is usually a numeric pain scale (NPS) up to 4/10.

Exclusion criteria included cauda equina syndrome (pluriradicular neurological involvement of the perineum and lower limbs without spinal cord involvement but with cauda equina compression) as well as motor deficit of lower limbs (motor strength ≤ 3/5 on MRC (Medical Research Council) scale) which constitutes a surgical emergency. Moreover, patients with migrated/excluded hernias, with a history of spine surgery or vertebral fracture, with contraindications of non-steroidal anti-inflammatory drugs (NSAID), corticosteroids, or epidural injection, and pregnant women were also excluded.

Participants were included during their hospitalisation by a hospital practitioner. Radicular pain was confirmed through clinical examination (straight leg raise test, Schober index, impulsivity, etc.) and after seeing MRI with objective nerve compression.

(c) Intervention

Lumbar tractions were performed using an automated table of continuous tractions in supine position knee on a cushion for 15 min per day. The traction force, empirically determined based on our clinical experience, was initially set a 15 kg for the first session and were progressively increased. The protocol was as follows: day 1; 15 kg, day 2; 18 kg, day 3; 21 kg, day 4 and 5; 24 kg. All the people were positioned in a supine position with a pillow under their knees. The protocol planned 3 to 5 tractions sessions. To not increase hospitalisation duration, 3 traction sessions were the most often performed.

Medical treatment was prescribed as needed by the treating physician (like intravenous NSAIDs or steroid treatments). At least two epidural injections were administered by physicians in charge of the participants. For epidural injections, 5 millilitres of corticosteroids and 10 mL of injectable physiological serum were injected into the sacrococcygeal hiatus. After that, people lay down for one hour. All treatments were delivered by nurses and was stalked following the hospital’s procedures. Physiotherapy was provided by a physiotherapist after a medical prescription.

Data was collected at inclusion by the treating physicians in charge of the participant on the fifth day, and at one and three-month follow-ups by unity practitioner via phone-call. Collected data at inclusion were demographics data (age, gender, activity, number of sick leave days due to radicular pain), radicular pain characteristics (radicular pain duration and localization, herniated disc position on MRI), medical treatment performed before inclusion (including epidural injection), radicular and lumbar Numeric Pain Scale (NPS) and Straight Leg Rising test (SLR test). The Numeric Pain Scale was used to collect pain levels orally on a scale of 0 (no pain) to 10 (the worst pain imaginable).

During hospitalisation, we registered C reactive protein levels at inclusion, use and tolerance of analgesics and anti-inflammatories treatments, epidural injections, and numbers of lumbar tractions. After five days of hospitalisation, participants had a new evaluation concerning radicular and lumbar Numeric Pain Scale, SLR test, and global satisfaction. At one and three months, participants were contacted by phone to register lumbar and radicular NPS, analgesics and anti-inflammatories consumption, work activity and/or necessity of work adaptation, indication of lumbar surgery, and their satisfaction regarding treatment efficacy and pain diminution.

At the end of the hospitalisation, all persons received the same prescription to carry out physiotherapy sessions with a liberal physiotherapist with the following instructions: learning spinal economics techniques, learning how to tilt the pelvis, work on locking the pelvis and proprioceptive work, passive and active muscle stretches of the hamstrings, rectus anterior, pyramidal and psoas, muscle strengthening of the spinals, abdominals mainly in endurance and quadriceps and hamstrings mainly in power, learning self-exercises for stretching and muscle strengthening.

(d) Outcome measures

The primary outcome was the participant proportion difference between the two groups with a minimum of 25% improvement of radicular pain at one month follow-up. Secondary outcomes were radicular NPS improvement of at least 25% at three months, lumbar NPS decrease of at least 25% at one and three months, duration of sick leave, SLR test improvement of at least 25% at five days, indication of a spinal surgery in the three months follow-up, decrease in analgesics use (decrease in one level), participants’ global satisfaction (estimated with a questionnaire on a scale from 0 (unsatisfied) to 100 (very satisfied)), and adverse events occurrence at five days, one month, and three months.

(e) Data analysis

The number of subjects required was calculated for the primary endpoint using the following assumptions: 80% of participants were expected to achieve an improvement of at least 25% on the radicular numeric pain scale at 4 weeks in the traction group, while in the medical group, 65% were expected to achieve the same level of improvement at 4 weeks. With an alpha risk of 5% and a power of 90% and a two-sided test, the nQuery software (nQuery Version 7.0. Sample Size and Power Calculation. “Statsols” (Statistical Solutions Ltd), Cork, Ireland) estimated that 185 participants per group were needed.

In the initial protocol, 310 participants were planned to be included. Five amendments have been added to the protocol, mainly concerning an extension of the inclusion period with the increase in person number to 426 compared to the initial study design. This was decided to realise a superiority study with bilateral analysis. The amendments also concern the addition of a per-protocol analysis and the removal of a non-inclusion criterion concerning BMI (Body Mass Index) which was, ultimately, not a limiting factor. The number of subjects required was therefore increased to 426 (213 per group). Missing data were not imputed; it was not foreseen in the protocol. Subgroup analysis was not prespecified.

Statistical significance was set at *p* < 0.05. All statistics were performed with SAS software version 9.4 (SAS Institute Inc., Cary, NC, USA). 

## 3. Results

### 3.1. Study Population

**Figure 1:**
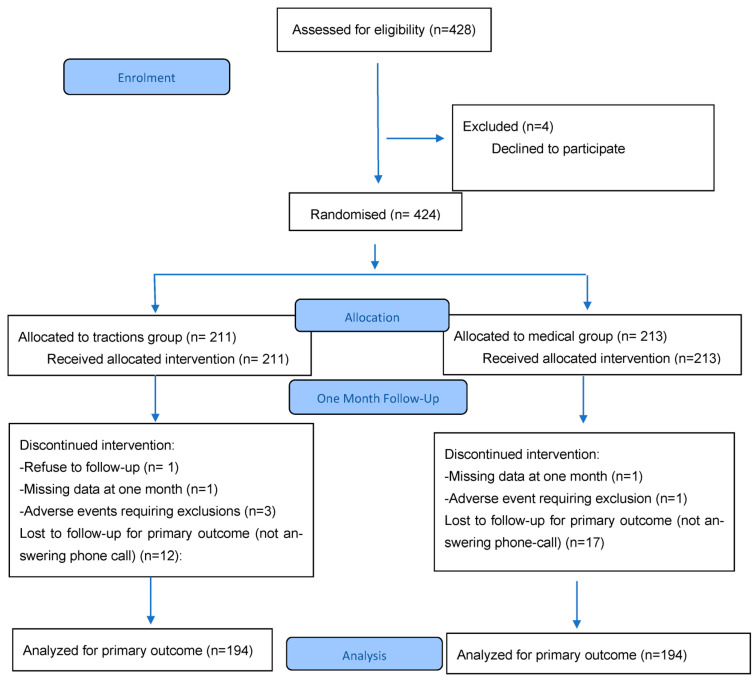
Flow chart.

Initially, 428 participants signed an informed consents but four withdrew before randomisation. A total of 424 participants were included until 2021 and were randomised in two groups: 211 (49%) in tractions group and 213 (50%) in medical group, as seen in flow of participants (Figure 1).

The two groups were similar at baseline. Baseline characteristics are summarised in Table 1.

### 3.2. Received Treatments

The treatments received by the participants during hospitalisation are summarised in Table 2.

### 3.3. Primary Outcome

For the primary outcome, the Intention-To-Treat (ITT) analysis, conducted on 388 participants, showed that 62% (120/194) of participants in the traction group and 51% (98/194) in the medical group experienced at least a 25% reduction in radicular pain, as measured by the Numeric Pain Scale. The likelihood of improvement was approximately 1.6 times higher in the traction group [OR 1.589 (95% IC 1.061–2.379, *p* 0.024)].

The Per-Protocol (PP) analysis, performed on 383 participants, found that 62% (117/189) of participants in the traction group and 51% (98/194) in the medical group showed improvement. The traction group was about 1.6 times more likely to experience improvement then medical group [OR 1.592 (95% IC 1.060–2.391), *p* 0.025].

**Figure 2:**
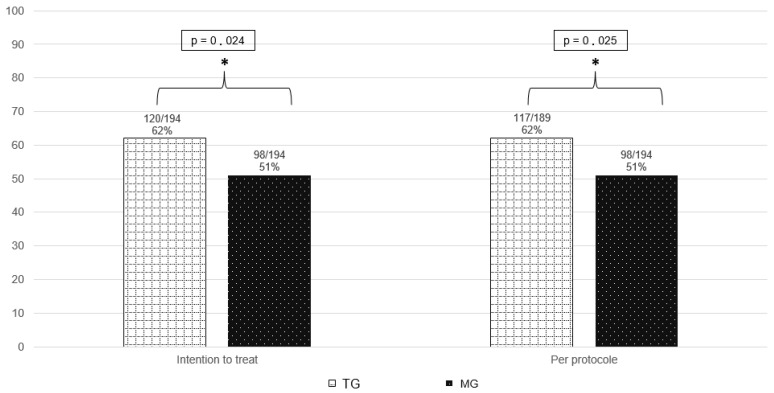
Primary outcome. * highlight a statistically significant difference.

There was a statistically significant difference in favour of the tractions group, as seen in Figure 2 and Table S1.

Participants who required surgery before one month were considered not improved by 25% on radicular NPS (*n* = 10 in tractions group and *n* = 7 in medical group). Mean radicular NPS in tractions group was 35.23 [SD (standard deviation) 28.85] and 40.64 (SD 30.85) in medical group.

In addition, we performed a sensitivity analysis with the maximum bias hypothesis: loss of follow-up participants or with missing data at one month have been considered as a treatment failure. We analysed the 424 included persons in ITT: 120/211 participants (57%) in tractions group and 98/213 participants (46%) in medical group. This analysis confirmed other results, showing that participants were 1.5 times more likely to experience improvement with the association of lumbar tractions [OR 1.545 (95% IC 1.036–2.312), *p* 0.025].

### 3.4. Secondary Outcomes

Secondary outcomes are summarised in Table 3 and Figure S1.

We have also collected participants’ satisfaction. There was a statistical difference between the two groups. At five days of hospitalisation, the global satisfaction in tractions group was 72/100 (SD 24.5) versus 67/100 (SD 27.7) in medical group (*p* = 0.067). At one month, participants’ satisfaction was 65/100 (SD 32.3) in tractions group and 59/100 (DS 30.8) in medical group (*p* = 0.018). At three months, global satisfaction was 69/100 (SD 33.8) in tractions group and 61/100 (SD 32.4) in medical group (*p* = 0.012).

### 3.5. Tolerance

Nine participants declared adverse effects: 7/211 persons in tractions group (3%) and 2/213 persons in medical group (1%) without statistical difference between them [OR 3.610 (95% IC 0.677–36.018), *p* = 0.104].

In the traction group, three participants experienced secondary motor impairments (1.4%). Two of these were transient: one was resolved surgically, and the other resolved spontaneously. The third participant still present a minor motor deficit (graded 4/5 on the Medical Research Council muscle strength scale), with no surgical indication. These cases were declared as serious adverse events at pharmacovigilance. However, due to their limited number and minimal long-term consequences, no statistical analysis was conducted or requested by pharmacovigilance authorities. One participant presented a contralateral radicular pain after the third traction, and three participants could not bear the traction protocol (7/211 participants: 3%). In medical group, one person presented a secondary motor impairment, and one person presented a spondylodiscitis (2/213 participants: 1%).

### 3.6. Subgroup Analysis

**Figure 3:**
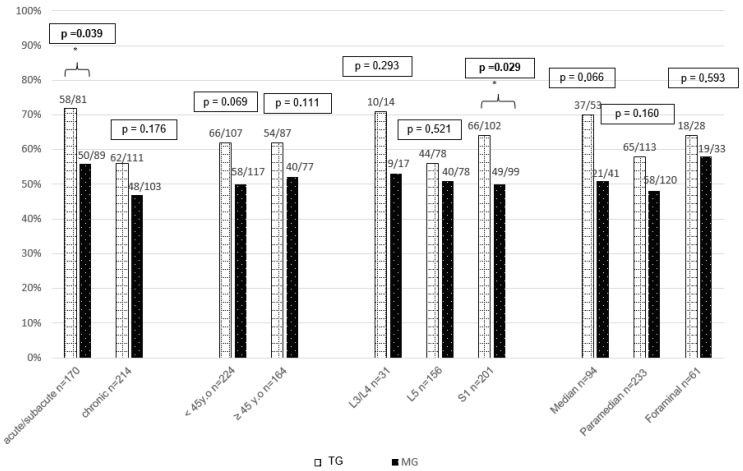
Subgroup analysis. * highlight a statistically significant difference.

To target a more specific population, we performed a subgroup analysis with clinically relevant criteria resumed in Figure 3 and Table S2. Lumbar tractions seem to be more effective in acute lumbar radicular pain and in S1 radicular pain with a significant difference.

## 4. Discussion

Results of this trial demonstrate the superiority of these particular lumbar tractions in association with medical treatment for a selected population experiencing radicular pain and a concordant disc herniation at one month follow-up. Furthermore, secondary analysis revealed an improvement in the return-to-work time.

To our knowledge, this is the largest superiority study in the literature evaluating these specific lumbar tractions efficiency in radicular pain secondary to disc herniation. We chose purposely to have stringent inclusions criteria and a control population to minimise selected bias inherent of this open-label trial. Proving and comparing the overall effectiveness of lumbar tractions is difficult due to the multitude of existing protocols in the literature: prone or supine [16], continuous or intermittent, mechanical or manual. This explains the divergent results found in the medical publications. The most common modalities are continuous or intermittent, mechanical or auto-tractions. There is no proof of superiority of one over the other modalities [17,18].

The empirical use of tractions has been going on for several decades, as evidenced by Cyriax’s 1950 paper, and efficacity was explained by an increase in the interval between vertebral bodies, enlargement of the space into which the protrusion must recede, and tautening of the joint capsule [13]. Decades later, in 2005, the tractions efficacy explanation is almost the same [12]. This prospective, randomised, and controlled trial included 46 participants. It compared a traction group (physiotherapy and tractions, undetailed protocol) and the control group (physiotherapy alone: ultrasound, hot pack and diadynamic currents). The size of the herniated disc was evaluated by CT before and after the tractions, showing a decrease in the hernia size with a significant difference.

In 2000, an American team studied the SLR test amelioration according to traction strength. They showed a decrease in SLR test pain intensity with traction strength from 30% to 60% of body weight [19].

A study on 45 participants with radicular pain due to disc herniation confirmed on MRI compared SLR test amelioration in function of tractions strength: one fifth, one third, or half body weight [20]. There was a significant amelioration of SLR test between inclusion and immediately after tractions in all groups. There was also a significant amelioration on radicular pain between inclusion and after 50% body weight tractions. No superiority of one of these modalities over the other has been proved. Our clinical experience show that a gradual increase was better tolerated than an important strength from the beginning.

We acknowledge that this study has some limitations, including the lack of control over post-hospitalisation recommendations. At the end of their hospitalisation, all participants received a formal physiotherapy prescription. It was difficult to evaluate bias weight inherent of this physiotherapy in the trial results, due to different physiotherapists practices, even with the same prescription. Participants’ follow-up was performed via phone, with all the concomitant difficulties and potential self-reporting bias. Moreover, we noticed that participants were more satisfied in tractions group, maybe greatly due to placebo effect of lumbar tractions with the hospital physiotherapist. Because of this study design, this placebo effect cannot be quantified. Another limitation to our study was the blind absence due to the open label-design. The lack of blinding in our study may amplify the analgesic effect through a placebo response in participants receiving traction, compared to those receiving only medical treatment. A solution to minimise it would have been to perform tractions without weight for participants randomised to the medical group alone. Nevertheless, this potential bias was addressed by selecting a primary outcome that is both reproducible and clinically relevant.

Some data that would have been interesting to analyse were not collected, such as weight, height, BMI, traction force for all participants, and their functional capacities. One of our major limitations was the absence of ODI (Owestry Disability Index), due to the lack of use in our clinical practice at the time of the study design. A choice was made to use the back-to-work/usual activity instead.

In 2021, Kotb et al. had similar inclusion criteria to our study (radicular pain and concordant disc herniation on MRI) and similar outcomes (lumbar and radicular pain, functional capacity) [21]. In this study, 48 participants were randomised in 4 groups: 12 persons received CT-guided transforaminal injections, 12 persons received tractions every day during one month with an automatic table with flexed knees for 15 cycles of tension and relaxation, 12 persons received both treatments, and 12 persons received only analgesics treatments. Between inclusion and one month, participants with tractions associated with CT-guided transforaminal injections were significatively improved concerning functionals abilities.

However, this study only performed intragroup comparison and cannot conclude any superiority of one modality over another.

A second study compared lumbar tractions and transcutaneous nerve stimulation [22]. Inclusions criteria were similar; lumbar and radicular pain with disc herniation or protrusion on imaging. Results showed a superiority of tractions but on a small population of 44 participants and their control group was not relevant; transcutaneous nerve stimulation is not a recommended treatment in lumbar radicular pain.

The literature poorly describes adverse events due to their rarity. Thackeray and al. reported some increase in spinal stiffness, lumbar pain, or radicular pain but without statistical differences between both groups (tractions and physiotherapy versus physiotherapy alone) [23]. In our study, adverse events were uncommon and mostly concerned an increase in motor impairment apparition, lumbar, and radicular pain, like those described in the literature.

Another interesting result of our study is the impact of tractions on the mean back-to-work delay between the two groups. There were significantly more people back-to-work in the tractions group than the medical group. Most of literature data concerning this aspect were available in the surgical area; most of the time, the aim was to prove the superiority of mini-invasive techniques. In one study, the mean back-to-work-delay was between six and twenty days depending on work type [24]. Low back pain and radicular pain are a significant public health issue in France, with a considerable socio-professional cost, including prolonged sick leave and increased use of medical care. This cost is estimated to 1.5% of European Gross Domestic Product [25]. Our study highlights the faster back-to-work delay in the tractions group. This socio-professional impact should therefore not be neglected.

Our subgroup analysis suggests that lumbar tractions are more effective for participants with acute or subacute radicular pain of S1 region, which seems to be consistent with a study showing that the residual effect of lumbar tractions is more important on lower lumbar discs [26]. Moreover, studies have shown that lumbar tractions are more effective when applied in recent cases [27,28]. However, no statistical difference was found to suggest greater efficiency of lumbar tractions in younger people. A potential confusing factor in elderly people could be the predominance of degenerative impairments, for which tractions have proved to have no impact on this type of lesion [29].

## 5. Conclusions

We can propose that lumbar tractions, when combined with medical treatment, may be beneficial given the demonstrated synergy between these two associated treatments and the low rate of adverse events. People must be selected with precaution and must present radicular pain due to disc herniation concordant with their symptoms. Lumbar tractions must be considered as an additional therapeutic weapon for a target population. Additional analysis is needed, especially on the young population with recent radicular pain in the S1 territory.

## Figures and Tables

**Table 1:** Baseline characteristics.

		TG ^1^ (N = 211)	MG ^2^ (N = 213)	Total (N = 424)
Female sex (%)		96 (45)	89 (42)	185 (44)
Mean age (years and SD)		43·7 (±12.63)	42·9 (±11.59)	42·7 (±12.24)
Professional activity (%)	Working patients	60 (28)	69 (32)	129 (30)
	Sick leave	104 (49)	103 (48)	207 (49)
	Students, retired, unemployed	47 (22)	41 (19)	88 (21)
Duration (%)	>3 months	120 (57)	115 (54)	235 (55)
	1–3 months	51 (24)	55 (26)	106 (25)
	<1 month	40 (19)	43 (20)	83 (20)
Previous injections (%)		108 (51)	94 (44)	202 (48)
Mean NPS radicular pain (SD)		62·81 (±24.47)	59·08 (±23.47)	60·94 (±23.52)
Mean NPS lumbar pain (SD)		48·17 (±27.48)	47·29 (±28.07)	47·73 (±27.75)
Previous anti-inflammatory use (%)	NSAIDs ^3^	128 (61)	125 (59)	253 (60)
	Corticosteroids	89 (42)	87 (41)	176 (42)
Radicular pain (%)	L3/L4	15 (7)	18 (8)	33 (7)
	L5	86 (41)	84 (39)	170 (40)
	S1	110 (52)	111 (52)	221 (52)
Disc herniation position (%)	Paramedian	122 (58)	130 (61)	252 (59)
	Median	60 (28)	46 (22)	106 (25)
	Foraminal	29 (14)	37 (17)	66 (16)

^1^ TG: tractions group; ^2^ MG: medical group; ^3^ NSAIDs: Non-Steroidal Anti-Inflammatory Drugs.

**Table 2:** Received treatments during hospitalisation.

		TG ^1^ (211)	MG ^2^ (213)	Total (424)
Traction	5 tractions	41	-	41
	4 tractions	84	-	86
	3 tractions	77	-	80
	2 tractions	7	-	7
	1 traction	2	-	2
NSAIDs ^3^		121	126	247
Cortico-steroids		37	39	76
Injections	3 injections	50	49	99
	2 injections	133	123	256
	1 injection	3	6	9
	Missing data	25	35	60
Analgesics treatments (strongest)	1	32	42	74
	2	128	120	248
	3	42	44	86
	Missing data	9	7	16

^1^ TG: tractions group; ^2^ MG: medical group; ^3^ NSAIDs: Non-Steroidal Anti-Inflammatory Drugs.

**Table 3:** Secondary outcomes.

	TG	MG	*p*	OR [95% Confidence Interval]
Diminution at least 25% for radicular NPS at 3 months (%)	114/181 (63)	96/179 (54)	0.072	1.471 [0.966; 2.241]
Diminution at least 25% for lumbar NPS at one month (%)	99/194 (51)	98/194 (51)	0.919	1.021 [0.686; 1.520]
Diminution at least 25% for lumbar NPS at 3 months (%)	91/181 (50)	77/179 (43)	0.168	1.339 [0.884; 2.029]
Back-to-work delay at one month (%)	30/88 (34)	14/87 (16)	0.008	2.682 [1.245; 6.013]
Back-to-work delay at 3 months (%)	44/88 (50)	23/87 (26)	0.002	2.766 [1.411; 5.528]
SLR test (%)	120/194 (62)	108/199 (54)	0.128	1.366 [0.914; 2.043]
Spinal surgery at one month (%)	10/194 (5)	7/194 (4)	0.621	1.451 [0.486; 4.592]
Spinal surgery at 3 months (%)	27/181(15)	34/179 (19)	0.327	0.748 [0.412; 1.349]
Analgesics diminution at one month (%)	55/157 (35)	55/144 (38)	0.632	0.873 [0.531; 1.434]
Analgesics diminution at three months (%)	69/157 (44)	53/144 (37)	0.240	1.345 [0.826; 2.197]

## Data Availability

The data presented in this study are available on request from the corresponding author.

## Supplementary Materials

The following supporting information can be downloaded at: https://www.mdpi.com/article/10.3390/jcm14155192/s1, Figure S1: Secondary outcomes; Table S1: Primary outcome: percentage of participants with at least 25% of amelioration on radicular NPS between inclusion and one month; Table S2: Subgroup analysis.

## Abbreviations

The following abbreviations are used in this manuscript:

BMI	Body Mass Index
CT	Computed Tomography
ITT	Intention To Treat
MG	Medical Group
MRC	Medical Research Council
MRI	Magnetic Resonance Imaging
NPS	Numeric Pain Scale
NSAIDs	Non-Steroidal Anti-Inflammatory Drugs
ODI	Oswestry Disability Index
OR	Odd Ratio
PP	per protocol
SD	Standard Deviation
SLR test	Straight Leg Rising test
TG	Tractions group
