# Supplementary material for: Lumbar Tractions in Radicular Pain Caused by Herniated Disc: Randomised, Open-Label, Superiority, and Controlled Trial on 424 Participants
Source: J Clin Med. 2025 Jul 22;14(15):5192. doi: 10.3390/jcm14155192 (PMC12347135; doi:10.3390/jcm14155192)
Supplement: Supplementary file 1 [file jcm-14-05192-s001.zip › jcm-3707971-supplementary.pdf]

Table S1. Primary outcome:: percentage of participants with at least 25% of amelioration on radicular NPS between inclusion and one month.

	TG[5] (N = 194)	MG[2] (N = 194)	*p*	OR[3] [95% Confidence Interval]
Decrease of at least 25% on radicular Verbal Pain Scale between inclusion and one month—ITT [1] (%)	120 (62)	98 (51)	**0.024**	**1.589 [1.061; 2.379]**
Decrease of at least 25% on radicular Verbal Pain Scale between inclusion and one month—PP[4] (%)	117 (62)	98 (51)	**0.025**	**1.592 [1.060; 2.391]**

[1] ITT: intention to treat; [2] MG: medical group; [3] OR: odds ratio; [4] PP: per protocol; [5] TG: tractions group.

Figure S1. Secondary outcomes.

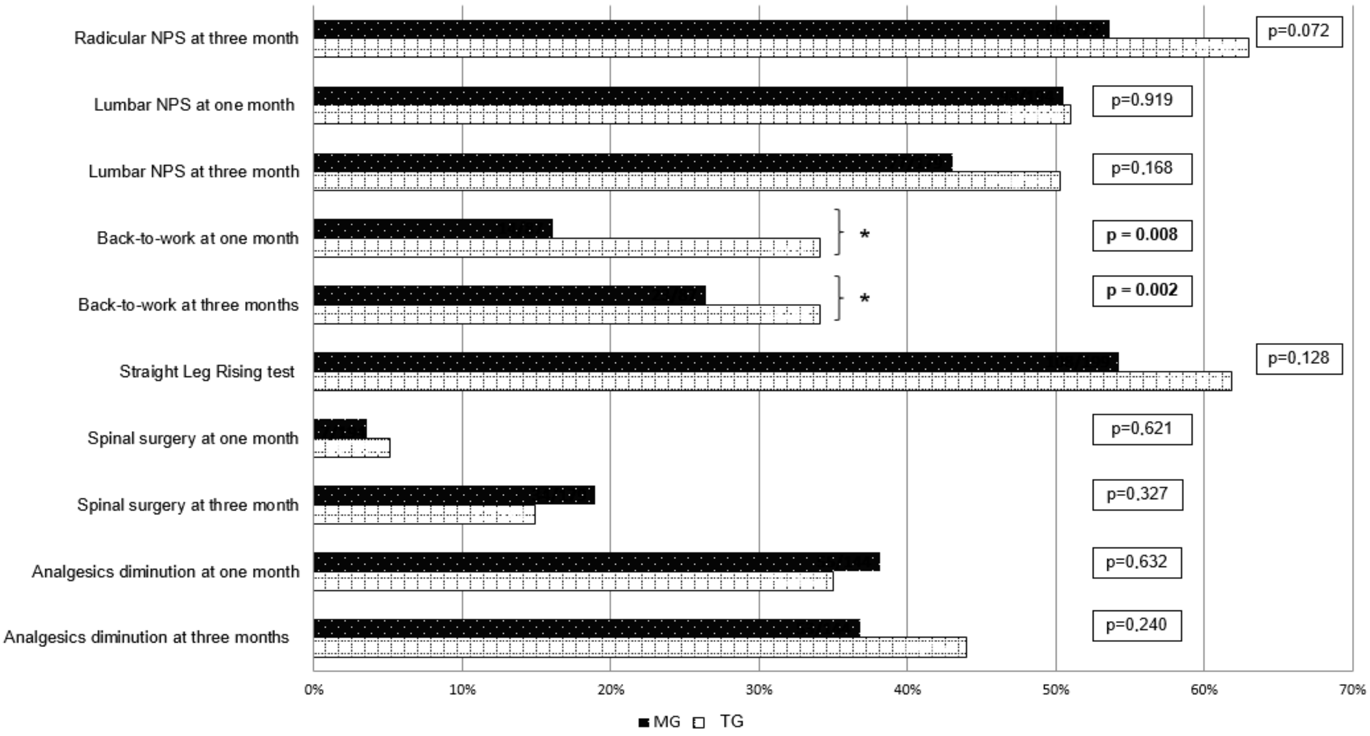

***highlight a statistically significant difference**

Table S2. Subgroup analysis.

	TG	MG	p	OR [1] [95% Confidence Interval]
Evolution period				
Acute (%)	29/33 (88)	22/36 (61)	0.011	3.515 [1.182; 10.452]
Subacute (%)	29/48 (60)	28/53 (53)	0.443	1.363 [0.618; 3.005]
Chronic (%)	62/111 (56)	48/103 (47)	0.176	1.450 [0.846; 2.485]
Age				
<45 y.o [2] (%)	66/107 (62)	58/117 (50)	0.069	1.638 [0.962; 2.788]
≥45 y.o (%)	54/87 (62)	40/77 (52)	0.111	1.514 [0.812; 2.821]
Radicular topography				
L3/L4 (%)	10/14 (71)	9/17 (53)	0.293	2.222 [0.496; 9.962]
L5 (%)	44/78 (56)	40/78 (51)	0.521	1.229 [0.655; 2.309]
S1 (%)	66/102 (64)	49/99 (50)	0.029	1.871 [1.062; 3.294]
Disc herniation position				
Median (%)	37/53 (70)	21/41 (51)	0.066	2.202 [0.943; 5.142]
Paramedian (%)	65/113 (58)	58/120 (48)	0.160	1.448 [0.863; 2.428]
Foraminal (%)	18/28 (64)	19/33 (58)	0.593	1.326 [0.470; 3.739]

[1] OR: Odd Ratio; [2] y.o: years old.
